# mutTCPdb: a comprehensive database for genomic variants of a tropical country neglected disease—tropical calcific pancreatitis

**DOI:** 10.1093/database/bay043

**Published:** 2018-07-24

**Authors:** Garima Singh, Basharat Bhat, M S K Jayadev, Ch Madhusudhan, Ashutosh Singh

**Affiliations:** 1 Department of Life Sciences, School of Natural Sciences, Shiv Nadar University, Greater Noida, Uttar Pradesh, India; 2 Department of Surgical Gasteroenterology, Osmania General Hospital, Hyderabad, Telangana, India

## Abstract

Tropical calcific pancreatitis (TCP) is a juvenile, non-alcoholic form of chronic pancreatitis with its exclusive presence in tropical regions associated with the low economic status. TCP initiates in the childhood itself and then proliferates silently. mutTCPdb is a manually curated and comprehensive disease specific single nucleotide variant (SNV) database. Extensive search strategies were employed to create a repository while SNV information was collected from published articles. Several existing databases such as the dbSNP, Uniprot, miRTarBase2.0, HGNC, PFAM, KEGG, PROSITE, MINT, BIOGRID 3.4 and Ensemble Genome Browser 87 were queried to collect information specific to the gene. mutTCPdb is running on the XAMPP web server with MYSQL database in the backend for data storage and management. Currently, the mutTCPdb enlists 100 variants of all 11 genes identified in TCP, out of which 45 are non-synonymous (missense, nonsense, deletions and insertions), 46 are present in non-coding regions (UTRs, promoter region and introns) and 9 are synonymous variants. The database is highly curated for disease-specific gene variants and provides complete information on function, transcript information, pathways, interactions, miRNAs and PubMed references along with remarks. It is an informative portal for clinicians and researchers for a better understanding of the disease, as it may help in identifying novel targets and diagnostic markers, hence, can be a source to improve the strategies for TCP management.

Database URL: http://lms.snu.edu.in/mutTCPDB/index.php

## Introduction

Tropical Calcific Pancreatitis (TCP) is a juvenile, non-alcoholic idiopathic chronic pancreatitis, prevalent in the tropical regions with unknown aetiology and is defined under the idiopathic category of TIGAR-O (i) toxic-metabolic, (ii) idiopathic, (iii) genetic, (iv) autoimmune, (v) recurrent and severe acute pancreatitis or (vi) obstructive) classification system (1). In 1980, juvenile tropical pancreatitis was described by the authors in the journal Lancet (2). Then, in the same year, Tan *et al*. describedspecifically ‘TCP’, for the first time (3). TCP is associated with clinical manifestations that include severe abdominal pain, pancreatic calculi, steatorrhoea, bilateral parotid enlargement, cyanotic hue of lips and fibrocalculous pancreatic diabetes (FCPD). FCPD is the characteristic feature of TCP and a distinct form of diabetes (Ketosis resistant) as described by World Health Organization (4). There are also evidences of head mass or tumors observed in 30–75% of TCP patients as reported by the authors (5). Hence, TCP can lead to malignancy in late stage. Morphologically, TCP is characterized by pancreatic ductal dilation, large dense calculi (6), pancreatic atrophy and fibrosis (7–9). Zuidema, in 1959, first reported pancreatic calculi and symptoms of malnutrition in many patients (10). Down the line, most studies were reported from Indian subcontinent. However, reports were also recorded from other tropical countries in Asia (11) (Malaysia, China, Japan, Bangladesh (12)), Africa (13) (Uganda, Nigeria (14)), South America (Brazil) and Mexico (15). A survey on chronic pancreatitis (CP) in Asia-Pacific region found that 70% of CP patients in India and China fall in the criteria of TCP (16). The symptoms of TCP overlap with other types of pancreatitis (alcoholic, hereditary and drug induced), therefore there is still a lack of strategic clinical management of this disease.

Extensive calcification of exocrine pancreas, large stones and dilated pancreatic duct are some of the phenotypic diagnostic markers for TCP in non-alcoholic patients. But there are no potential molecular/genetic biomarkers identified till now, because of the lack of objective knowledge about the pathophysiology involved in TCP. The treatment for TCP is mostly surgical in the form of removal of all intraductal stones. Chromogenic techniques (17) such as endoscopic retrograde cholangiopancreatography, computed tomography scan, trans-abdominal ultrasonography, and so on can clinically diagnose the pervasive calcification in the exocrine pancreas.

Our database, ‘mutTCPdb’ contains single nucleotide variants (SNVs) present in genes, so far identified in TCP patients (patients with non-alcoholic chronic pancreatitis). The genes defined in the database are; extracellular calcium sensing receptor (CASR), cystic fibrosis transmembrane conductance regulator (CFTR), chymotrypsin-C, cathepsin B (CTSB), pancreatic secretory granule major glycoprotein GP2 (GP2), protease, serine, 1 (trypsin 1) (PRSS1), protease, serine, 2 (trypsin 2) (PRSS2), regenerating islet-derived 1 alpha (REG 1 A), serine peptidase inhibitor, Kazal type 1 (SPINK1), transcription factor 7-like 2 (TCF7L2) and carboxypeptidase A (CPA1). These genes have variants that are associated with non-alcoholic chronic pancreatitis in the tropical regions. There is a research article, which negates the association of angiotensin converting enzyme (ACE) gene to TCP, based on direct DNA sequencing studies of ACE gene (18). Recently, an association between CLDN2-MORC4 and PRSS1-PRSS2 locus variants have been established with TCP (19). A variant C677T in 5, 10-methylenetetrahydrofolate reductase was found to be associated with tropical chronic pancreatitis patients from north Indian population (20).

In general, ‘mutTCPdb’ was created with an objective to organize the sparsely distributed data on genetic variants studied so far in TCP. This manually curated comprehensive database is an integrated and updatable mutations resource for clinicians and scientists working on TCP.

## Materials and methods

###  Data collection

TCP is a neglected tropical disease, therefore limited information is available. Initially, the PubMed (http://www.ncbi.nih.gov/pubmed) was searched with terms ‘TCP’ [Title/Abstract/Text Word], ‘Tropical Pancreatitis’ [Title]/Abstract/Text Word], ‘Tropical Chronic Pancreatitis’ [Title/Abstract/Text Word] and ‘Chronic calcific non-alcoholic pancreatitis’ [Title/Abstract/Text Word]. Consequently, around 500 published articles since 1980 were enlisted for the above queries till July 2017. Additionally, the NCBI portal (https://www.ncbi.nlm.nih.gov/), Ensemble Genome Browser 87 (http://asia.ensembl.org/index.html) and UNIPROT (http://www.uniprot.org/) sources were also used for supplementary information.

### Database organization

**Figure 1.**
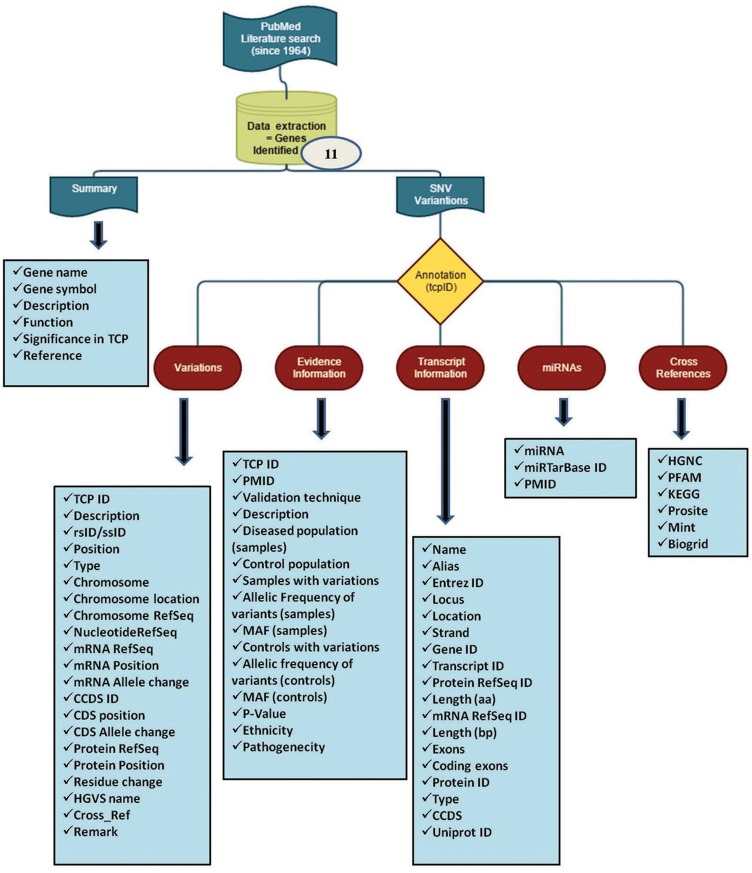
Flowchart of work plan for mutTCPdb. The database consist of (1) extraction of data from literature that includes complete information about the variants studied so far in TCP and (2) annotating the variants manually and categorizing them in various sublevels to define each variant completely.

After retrospectively screening the literature, SNVswere extracted from published articles and the data were organized in various levels. Each variant in a specific gene was annotated with an ID (e.g. tcp8461). The initial query starts with Gene_symbol (e.g. CASR, CFTR), Entrez_ID (e.g. 846) or Gene_ID (e.g. ENSG00000036828) and then divided into the following headings **(**Figure 1).

#### (A) Summary

This section provides the information of every gene associated with TCP and studied for variants. The significance of the respective gene to be involved in TCP was curated from publications on PubMed (https://www.ncbi.nlm.nih.gov/pubmed).

#### (B) SNV information

The information for SNVs in respective genes is subdivided in the following descriptors:

##### 1. Variants

The variant information of each single nucleotide variant (SNV) in a gene is divided in two categories: (i) databases-derived information and (ii) literature-derived information.

‘Literature-derived information’ segment has the curated information (genomic data) for each variant available in the published article. If the data for a specific variable (e.g. Chromosome Refseq_ID, Nucleotide Refseq_ID etc.) were not available in the literature, the column was left with a hyphen (-).

For each variant, the ‘database-derived information’ segment has complete transcript information available on dbSNP database build 150 (http://www.ncbi.nlm.nih.gov/SNP/) with respect to human genome assembly, GRCh38.p7. Every variant is defined and attached with a ‘remark’ column to simplify the user query. Besides, cross reference (Cross_Ref) column list other diseases, if associated with that particular variant.

##### 2. Evidence information

This section contains all the relevant information from the published literature about variants identified in that particular gene.

##### 3. Transcript information

The data in this table include, gene_name, Alias, Entrez_ID, Locus, Strand, Gene_ID, Transcript_ID, Protein RefSeq_ID, Length(aa), mRNA RefSeq_ID, Length(bp), Exons, Coding exons, Protein RefSeq_ID, Type, CCDS, Uniprot_ID. The information respective to each variable is extracted from NCBI portal and Ensemble Genome Browser 87.

#### (C) MicroRNA predictions

This tab includes validated MicroRNAs (miRNAs) targeting each TCP-related genes. The miRNA data weres obtained from miRWalk2.0 algorithm and miRTarBase database (21).

#### (D) Cross-reference

The miscellaneous identifiers for each gene are listed in this section for user reference.

## Web interface and data browser

**Figure 2.**
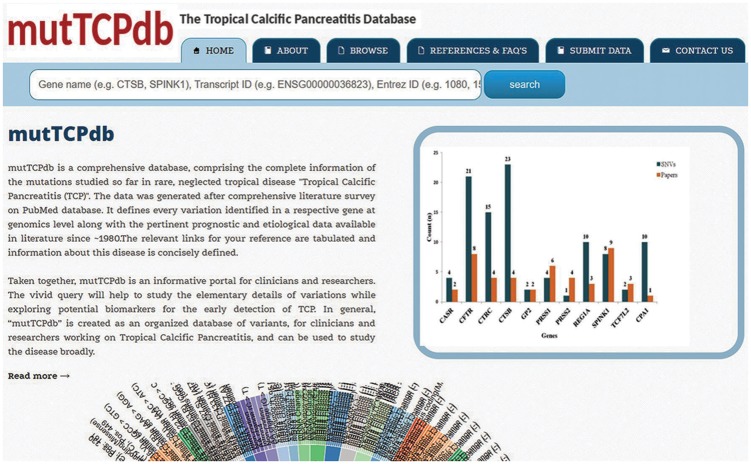
Web interface of mutTCPdb. The home page of database displaying an interactive wheel browser.

**Figure 3.**
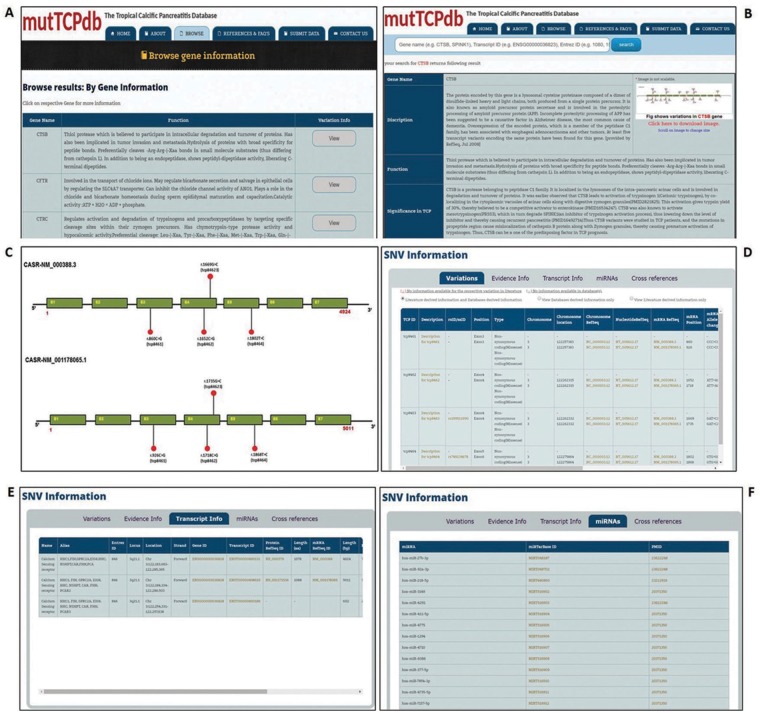
Data browsing in mutTCPdb. (**A**) ‘Browse’ tab gives the list of genes described in database. (**B**). Search mutTCPdb using a keyword (Gene Symbol). The results obtained after keyword search. (**C**) The graphical layout of SNPs identified in the query gene. (**D**) Details of variants identified in query gene. (**E**) Transcript information for each variant for the query gene. (**F**) Details of miRNAs associated with the query gene.

The web interface for mutTCPdb is uncomplicated, researcher friendly and interactive. mutTCPdb focuses on providing better navigation through individual sections to increase data discoverability. There are six tabs provided at the top of the interface (‘Home’, ‘About’, ‘Browse’, ‘Stats and FAQs’, ‘Submit Data’ and ‘Contact Us’) through which users can navigate and explore the required information. mutTCPdb is running on XAMPP web server with MYSQL database in the backend for data storage and management. Text query box is provided at the top of each page to search by Gene name, Entrez ID or Transcript ID related to every gene. The data can be accessed either by browsing through the predefined lists (provided at ‘Browse’ tab) or through search box. The snapshots of the database are illustrated in Figures 2 and 3.

Query processing scripts are written in PHP and Perl. The database is tested and works well with commonly available web browsers, such as Mozilla Firefox, Google Chrome, safari and Microsoft Internet Explorer.

There is a provision of submitting new data to mutTCPdb under the ‘Submit Data’ tab. The user can submit the data using an interactive form and then upload the file in any format. Once the data are submitted, the admin will verify the information from the references cited and also check for the duplicate entry in mutTCPdb. If the admin finds no discrepancy, the information will be uploaded and made available to the users. Additionally, mutTCPdb 1.0 will be updated regularly, every 24 months, with new data and novel features.

## Statistics and results

**Figure 4.**
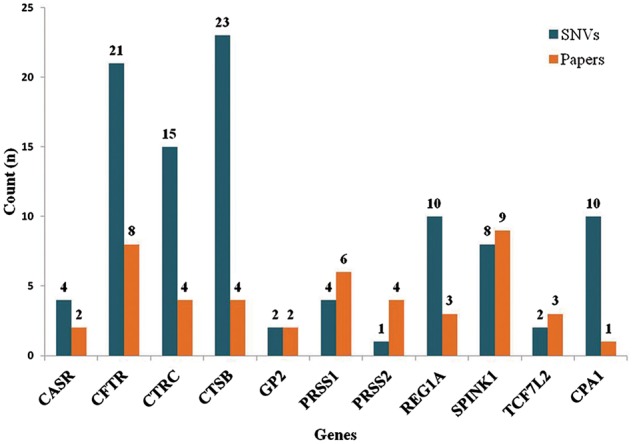
**Statistics (Part 1)**. The graph depicts number of variants identified for respective gene and also the number of published articles which were queried for each gene. There are 100 variants extracted from literature present in 11 genes.

**Figure 5.**
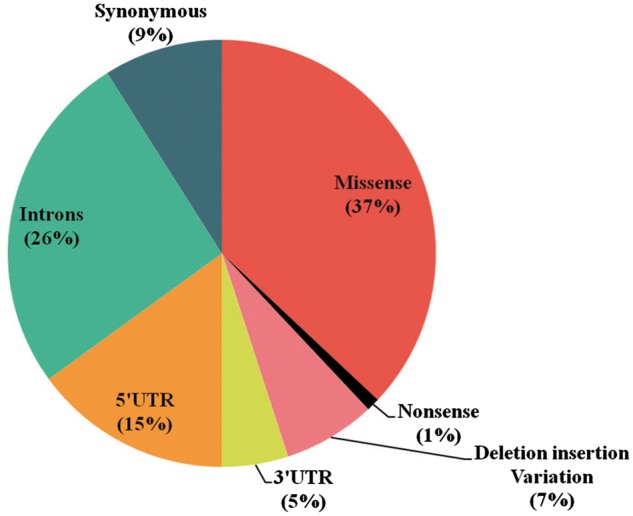
**Statistics (Part 2).** A. The pie chart depicts the percentage of types of variants defined in mutTCPdb.

The database (mutTCPdb) consists of 100 variants found within 11 genes studied so far associated with TCP patients (complete statistics is described in Figure 4). These variants **(**Figure 5) were further classified into missense (*n* = 37), nonsense (*n* = 1), deletions–insertion variants (*n* = 7) and synonymous variants (*n* = 9). Non-coding variants were subdivided as, 5′UTR (Untranslated regions) variants (*n* = 15), 3′UTR variants (*n* = 5) and intronic variants (*n* = 26). Deletion–insertion variants were positioned in introns, exons or in UTRs. We used GeneHancer to check whether the variants present in the non coding regions fall in gene enhancer regions or not (22). Out of all the genes in the database, we could only find seven variants in 5′UTR regions present in CTSB gene and two variants in TCF7L2 gene, to fall in enhancer regions (Table 1). Each variant was specifically extracted from a published article and linked with its population data. The ‘Description’ and ‘Function’ heading gives the information about the gene location, activity and role. The ‘Significance in TCP’ heading explains the rationale behind a gene to be considered as pathogenic in TCP. For example, the CPA1 gene has been included in database based on theresearch article by Witt et al., which describes the variants in CPA1 gene to be associated with TCP (23). Also, the article describes that the reduced activity of mutant CPA1 gene during experimental study might be due to reduced secretion of CPA1 as a result of misfolding in ER as stated in ‘Significance in TCP’ tab. Hence, the selected genes have been curated form published articles on the basis of variants present in that gene, respectively, identified in TCP patients.

**Table 1.** The table indicates the 5′UTR variants in CTSB and TCF7L2 genes with there corresponding Gene Enhancer IDs in the second column

TCP ID	Gene Hancer ID
tcp15084	GH08G011856
tcp15085	GH08G011856
tcp15088	GH08G011851
tcp15089	GH08G011851
tcp150810	GH08G011851
tcp150811	GH08G011851
tcp150813	GH08G011850
tcp69341	GH10G112997
tcp69342	GH10G113047

The first column has TCP variant ID’s for CTSB and TCF7L2 genes.

## Conclusion

mutTCPdb provides information regarding SNVs associated with TCP. This is first effort to manually resource all the variants present in the literature and make a comprehensive repository for TCP related SNV. Till now, neither the diagnosis nor the medications are specific for TCP because of overlapping symptoms with other forms of pancreatitis and lack of defined etiopathogenesis. ‘muTCPdb’ will help in characterizing the disease further by studying TCP at the molecular level and unfold the enigma hovering over the pathogenesis of TCP, which can rather be interpreted as an inflammation without a cause’. In future, mutTCPdb will be used to correlate present data with the next generation sequencing results like Exome and RNA sequencing, which can definitely enrich the current database. The first update of mutTCPdb will be released next year and further updates will be available after every 2 years.

## References

[1] Etemad B., Whitcomb D.C. (2001) Chronic pancreatitis: diagnosis, classification, and new genetic developments. Gastroenterology, 120, 682–707. 11179244 10.1053/gast.2001.22586

[2] Nwokolo C., Oli J. (1980) Pathogenesis of juvenile tropical pancreatitis syndrome. Lancet, 1, 456–459. 6102187 10.1016/s0140-6736(80)91001-6

[3] Tan C.T., Kannan P., Sng K.H. (1980) Tropical calcific pancreatitis. Med. J. Malaysia, 35, 150–154. 7266409

[4] World Health Organization. (1985) Diabetes mellitus. Report of a WHO Study Group. *Technical Report Series 727.* WHO, Geneva, pp. 1–113. 3934850

[5] Perumal S., Palaniappan R., Pillai S.A. et al (2013) Predictors of malignancy in chronic calcific pancreatitis with head mass. World J. Gastrointest. Surg., 5, 97–103. 23717745 10.4240/wjgs.v5.i4.97 PMC3664296

[6] Chari S., Jayanthi V., Mohan V. et al (1992) Radiological appearance of pancreatic calculi in tropical versus alcoholic chronic pancreatitis. J. Gastroenterol. Hepatol., 7, 42–44. 1543866 10.1111/j.1440-1746.1992.tb00932.x

[7] Khuroo N.S., Khuroo M.S., Khuroo M.S. (2010) Anomalous pancreaticobiliary ductal union in tropical calcific pancreatitis. JOP, 11, 18–24. 20065547

[8] Garg P.K. (2012) Chronic pancreatitis in India and Asia. Curr. Gastroenterol. Rep., 14, 118–124. 22327961 10.1007/s11894-012-0241-0

[9] Paliwal S., Bhaskar S., Chandak G.R. (2014) Genetic and phenotypic heterogeneity in tropical calcific pancreatitis. World J Gastroenterol., 20, 17314–17323. 25516642 10.3748/wjg.v20.i46.17314 PMC4265589

[10] Zuidema P.J. (1959) Cirrhosis and disseminated calcification of the pancreas in patients with malnutrition. Trop. Geogr. Med., 11, 70–74. 13659585

[11] Pitchumoni C.S. (1980) Juvenile tropical pancreatitis. Lancet, 1, 1028. 10.1016/s0140-6736(80)91464-6 6103351

[12] Khan A.A., Ali L. (1997) Tropical calcific pancreatitis and fibrocalculus pancreatic diabetes in Bangladesh. J. Gastroenterol. Hepatol., 12, S48–S52. 9195412 10.1111/j.1440-1746.1997.tb00458.x

[13] Sankalé M. (1984) Pancreatic pathology in black Africans (excluding diabetes). Med. Trop. (Mars), 44, 259–268. 6503680

[14] Yakubu A.M., Garg S.K., Umar B.A. et al (1984) Juvenile tropical pancreatitis syndrome in northern Nigeria: a case report. Ann. Trop. Paediatr., 4, 79–82. 6083749 10.1080/02724936.1984.11748313

[15] Uscanga L., Robles-Díaz G., Sarles H. (1985) Nutritional data and aetiology of chronic pancreatitis in Mexico. Dig. Dis. Sci., 30, 110–113. 3967558 10.1007/BF01308194

[16] Garg P.K., Tandon R.K. (2004) Survey on chronic pancreatitis in the Asia-Pacific region. J. Gastroenterol. Hepatol., 19, 998–1004. 15304116 10.1111/j.1440-1746.2004.03426.x

[17] Strobel O., Büchler M.W., Werner J. (2009) Surgical therapy of chronic pancreatitis: indications, techniques and results. Int. J. Surg., 7, 305–312. 19501199 10.1016/j.ijsu.2009.05.011

[18] Bhaskar S., Reddy D.N., Mahurkar S. et al (2006) Lack of significant association of an insertion/deletion polymorphism in the angiotensin converting enzyme (ACE) gene with tropical calcific pancreatitis. BMC Gastroenterol., 6, 42. 17163998 10.1186/1471-230X-6-42 PMC1762011

[19] Paliwal S., Bhaskar S. et al (2016) Association analysis of PRSS1-PRSS2 and CLDN2-MORC4 variants in nonalcoholic chronic pancreatitis using tropical calcific pancreatitis as model. Pancreas, 45, 1153–1157. 26784911 10.1097/MPA.0000000000000608

[20] Singh S., Choudhuri G. et al (2012) Association of 5, 10- methylenetetrahydrofolate reductase C677T polymorphism in susceptibility to tropical chronic pancreatitis in north Indian population. Cell. Mol. Biol. (Noisy-Le-Grand), 58, 122–127. 23273201

[21] Hsu S.D., Tseng Y.T., Shrestha S. et al (2014) MiRTarBase update 2014: an information resource for experimentally validated miRNA-target interactions. Nucleic Acids Res., 42, D78–D85. 24304892 10.1093/nar/gkt1266 PMC3965058

[22] Fishilevich S., Nudel R., Rappaport N. et al (2017) GeneHancer: genome-wide integration of enhancers and target genes in GeneCards. Database, 2017, 1–17. 10.1093/database/bax028 PMC5467550 28605766

[23] Witt H., Beer S., Rosendahl J. et al (2013) Variants in CPA1 are strongly associated with early onset chronic pancreatitis. Nat. Genet., 45, 1216. 23955596 10.1038/ng.2730 PMC3909499

